# The Referrer Matters. Musculoskeletal Surgical Conversion Rates: A Systematic Review With Meta-Analysis

**DOI:** 10.1177/11786329241304615

**Published:** 2024-12-03

**Authors:** Darryn Marks, Jasmine Pearce-Higgins, Taylor Frost, Joseph Fittock, Evelyne Rathbone, Wayne Hing

**Affiliations:** 1 Bond University, Robina, QLD, Australia; 2 Gold Coast University Hospital, Southport, QLD, Australia

**Keywords:** Musculoskeletal, orthopaedic surgery, physiotherapy, conversion rate, efficiency

## Abstract

**Background:**

Efficient musculoskeletal care is important for health services and society. Surgical conversion rates are a common measure of efficiency, yet normal values and the impact of referrer type are unclear. This information could assist musculoskeletal care, service benchmarking and redesign.

**Methods:**

A systematic review with meta-analysis was undertaken with PubMed, CINAHL and EMBASE databases searched from inception to 12th of October 2024, to identify studies from which musculoskeletal surgical conversion rates could be extracted. Data were categorised according to the professional group responsible for referral (all doctors, general practitioners, sports physicians, allied-health/physiotherapy-led screening services) and methodology used to define surgical conversion. Meta-analysis of pooled data was undertaken.

**Results:**

Twenty-eight studies with a combined total of 5358 patients were included. Pooled data revealed surgical conversion rates of 23% for referrals from all types of doctors (0.23, 95% CI 0.18-0.27), 28% from general practitioners (0.28, 95% CI 0.12-0.52), 61% from allied health physiotherapy-led screening services (0.61, CI 0.50-0.70) and 70% from sports physicians at (0.70, CI 0.64-0.75). A variety of methodological factors impacted surgical conversion rate reporting and heterogeneity.

**Conclusions:**

Musculoskeletal services seeking to improve efficiency through higher surgical conversion rates, should include sports physician and/or physiotherapy-led models of care for referral generation or management.

## Background

Musculoskeletal conditions are a leading cause of disability and persistent pain globally.^1^ As much of the world’s population becomes older, and increasingly sedentary, the prevalence of musculoskeletal disease is expected to increase, thereby amplifying demand for care^2,3^ and posing challenges for health and social care delivery. Surgical services will shoulder much of this burden, with rates of common procedures such as knee arthroplasty, predicted to increase 276% by 2030.^4^ While costly to governments and healthcare funders, timely surgical activity is important for society as it returns injured and disabled individuals to work and activity, thereby supporting productivity and societal economic stability. Furthermore, surgical departments such as orthopaedics are critical to the financial viability of hospitals, with 35% of elective surgery revenue and 23% of net hospital income resulting from elective surgery for musculoskeletal conditions.^5^ Thus, efficient musculoskeletal care is crucial for both health services and society.

A common measure used to assist organisational understanding of efficiency in surgical pathways is the surgical conversion rate (SCR), which represents the proportion of referrals that convert to surgery. This is calculated by dividing the number of surgical cases by the total number of cases seen and expressed as a decimal value or multiplied by 100 for a percentage value.^6^ A high SCR is associated with effective referral pathways, efficient utility of the surgical workforce^7^ and greater service revenue.^8^ In contrast, with a low SCR proportionally fewer referred patients require surgery. This may be associated with longer waiting times for patients and reduced organisational productivity, for example if a significant amount of surgeons’ time is consumed by non-surgical cases.

Given the importance to health organisations of understanding efficiency and demand for services,^9^ surprisingly little is known about SCRs for musculoskeletal referrals. Prior reviews have focused on orthopaedic waiting list management by professional groups such as physiotherapists in orthopaedic screening services (where patients who were referred to surgeons are instead seen by experienced physiotherapists), and reported that appropriately trained physiotherapists in these services can effectively triage, manage musculoskeletal referrals and improve SCR^10-13^ but the actual impact on SCRs has been less clear.^11^ The SCR has been estimated with a wide range of 25% to 91% by a prior review specifically of extended scope physiotherapy services^14^ and no meta-analysis has been conducted. While these findings highlight potential efficiency gains from interprofessional collaboration, several knowledge gaps continue to hinder service design; typical SCRs in orthopaedic/musculoskeletal settings are poorly understood, the impact of varying referral rules and pathways (based on professional groups) have not been clearly compared and methodological variables impacting SCR reporting have not been highlighted. Greater intelligence about SCRs could assist evaluation of referral processes, support benchmarking across services and aid efficient service redesign. Therefore, the primary aim of this review was to report surgical conversion rates for musculoskeletal disorders and quantify the impact on SCR of referrals from different professional groups. The secondary aim was to describe methodological factors that impact SCR reporting.

## Methods

A systematic review with meta-analysis was undertaken, following Preferred Reporting Items for Systematic Reviews and Meta-Analyses (PRISMA).^15^ The protocol was registered on Open Science Framework (OSF), Registration: osf.io/c7dkz/ DOI: 10.17605/OSF.IO/C7DKZ.

### Search strategy

The search strategy was developed for PubMed and the Polyglot Search Translator used for translation to other databases.^16^ A search was conducted on the 11th of November, 2021 and updated on the 12th of October 2024, in PubMed, CINAHL and EMBASE databases. Reference lists were also screened manually.

The final search for PubMed was ((ortho*[tiab] OR orthopedics*[Mesh] OR neurosurgery[tiab]) AND (musculoskeletal*[tiab]) AND (refer*[tiab] OR ‘referral and consultation’[Mesh] OR consultation[tiab] OR triage*[tiab] OR triage[Mesh]) AND (surg*[tiab] OR non-surgical[tiab])). Full search strategies appear as Supplemental Files.

### Eligibility

Eligibility criteria were defined using a PICOS model^17^: *Population*: adults with a musculoskeletal disorder referred for consultation with a surgeon; excluded were studies involving patients specifically from emergency and paediatric services. *Intervention*: consultation with a surgeon. *Comparator*: not applicable (as SCR rather than the outcome of an intervention was the intention of the review). *Outcome*: Surgical conversion rate reported or able to be extracted. Studies in which surgical data was unable to be isolated (eg, if combined with other procedures such as injection) were excluded. *Study type*: All study types, published in English with full text available. Studies in which participants were allocated to surgical or non-surgical care (rather than clinician and patients decision-making) were excluded.

### Screening and data extraction

Search results were imported into EndNote 21 (Endnote 21, Thompson Reuters, New York, NY, USA).^18^ Imported studies were screened independently by 2 authors (T.F and J.F) using SR accelerator,^19^ with a third (J.PH) providing consensus if needed, to reduce inclusion duplication and selector bias.^20^ Following removal of duplicates, titles and abstracts were screened to determine full texts for retrieval. Data from included studies were extracted and tabulated using Microsoft Excel and independently assessed for accuracy by 2 reviewers (T.F and J.PH). Screening and data extraction for the updated search was conducted by 2 authors (DM and WH) following the same processes. Data items included: study characteristics (authors, publication year, title, study design, country, setting), patient population (type/s of MSK condition and age of participants), service characteristics (type of service and profession/s who referred to the surgical service), methodological characteristics (timepoint/s and/or descriptor/s of when a patient was deemed to be surgical), surgical conversion data (number of referrals and number of surgical cases).

### Data synthesis and analysis

SSCR data extracted from included studies were organised into 4 different categories according to referral source: *1. Referrals from all doctors* for cohorts referred in the traditional way, by any doctor with referral rights, including General Practitioners (GPs), specialist physicians or surgeons. This category was assumed unless other referral process were stated; *2. referrals from GPs only; 3. referrals from a screening service*, comprising non-medical professionals such as allied health professionals and/or nurses; *4. Referrals from sports physicians*. Methodology used within each included study to deem a case as surgical, were categorised into 1. surgeon’s opinion of surgical need (*Opinion*), 2. patients listed and/or consented for surgery (*Listed*) and 3. patients who had received surgery (*Had*). Where relevant data were present but SCR not reported, SCR was calculated as the number of surgical cases divided by the number of patients or consultations. Referred musculoskeletal disorders were reported if the sample only pertained to 1, 2 or 3 conditions (eg, shoulder and knee conditions), with studies presenting data for more than 3 conditions described as mixed. Dichotomous outcomes were meta-analysed in R statistical software^21^ version 4.1.2, using the package *meta*. Proportions for the individual studies were pooled using random effects models and the inverse variance method. Heterogeneity was assessed using the *I*^2^ statistic, with low, medium and high represented by >25%, >50%, >75%, respectively,^22^ however as the metanalysis presents observational data, sources of potential heterogeneity are also explored.

### Critical appraisal

Two authors (DM and WH) independently appraised included studies using the Joanna Briggs Institute (JBI) Appraisal Tools for cross-sectional studies^23^ as consensus deemed this the most appropriate tool for appraising studies with respect to the SCR focus of this review. Discrepancies were resolved through discussion, or involvement of a third author if required. The checklist comprised 8 questions, which were assigned a value of 1 when scored as ‘yes’ and a value of 0, when scored as ‘no’ or ‘unclear’. Total scores out of 8 are recorded in Table 1 and a detailed breakdown of scoring for each included study appears as supplementary information.

**Table 1.** Summary of included studies.

Study	Country setting	Study design	Age (mean or other indicator)	Condition/s	Referrer	No. seen by orthopaedics/neurosurgery	No. deemed surgical	How deemed surgical	Appraisal ( /8)
Aiken and McColl^24^	Canada Public	Inter-rater reliability	(Arthroplasty service)	Hip/Knee	GPs	38	25	Surgical opinion	5/8
Ashmore et al^25^	Ireland Public	Retrospective audit	46	Knee	Physiotherapy/Screening Service	50	42	Had surgery	7/8
Bath et al^26^	Canada Private	Retrospective audit	51	Spine/hip/knee	Physiotherapy/Screening Service	98	30	Surgical opinion	5/8
Bernstein^27^	UK Public	Retrospective audit	(Not stated)	Mixed	Physiotherapy/Screening Service	455	318	Surgical opinion	3/8
Blackburn et al^28^	Australia Public	Retrospective audit/cohort	(Not stated)	Spine LBP	Physiotherapy/Screening Service	17^a^	9	Had surgery	6/8
Burn and Beeson^29^	UK Public	Prospective cost/service evaluation	(Not stated)	Mixed	Physiotherapy/Screening Service	38	27	Listed for surgery	5/8
Campagna-Wilson et al^6^	Canada Military	Survey/retrospective service evaluation	42	Mixed	Physiotherapy/Screening Service	102	44	Surgical opinion	7/8
					All doctors	119	36		
Candy et al^30^	UK Public	Retrospective cross-sectional	(Not stated)	Mixed	Physiotherapy/Screening Service	462	237	Had surgery	6/8
Goodman et al^31^	USA Military	Prospective service evaluation	(18+)	Mixed	All doctors	731	140	Had surgery	7/8
Gwynne-Jones et al^32^	New Zealand Public	Prospective cohort	67	Hip/Knee	Physiotherapy/Screening Service	143	118	Listed for surgery	7/8
Hattam^33^	UK Public	Retrospective cross-sectional	(Median 36-55)	Mixed	Physiotherapy/Screening Service	170	89	Had Surgery	7/8
Heywood^34^	UK Military	Prospective service evaluation	(Military personnel)	Spinal	Physiotherapy/Screening Service	25	15	Had surgery	4/8
Hourigan and Weatherley^35^	Ireland Public	Prospective service evaluation	47	Spine	Physiotherapy/Screening Service	14	6	Had surgery	5/8
Kara et al^36^	Turkey Public	Retrospective cohort	22	Mixed	Sports Physicians	155	110	Surgical opinion	7/8
							76	Had surgery	
Lyons et al^37^	Ireland Public	Prospective service evaluation	(Not stated)	Shoulder	Physiotherapy/Screening Service	201	56	Listed for surgery	6/8
							50	Had surgery	
Marks et al^38^	Australia Public	Prospective cost of illness	57	Shoulder	GPs	277	61	Had surgery	7/8
Mathieu et al^39^	Canada Public	Retrospective cross-sectional	61	Spine LBP	All doctors	500^a^	112	Surgical opinion	8/8
Mayman and Yen^40^	Canada Public	Prospective service evaluation	(Not stated)	Spine (thoracic or LBP)	All doctors	142^a^	27	Surgical opinion	6/8
Menzies and Young^41^	USA Public	Retrospective cohort	51	Mixed	Sports Physicians	118	80	Surgical opinion	7/8
Murphy et al^42^	Ireland Public	Retrospective service evaluation	45	Spine LBP	Physiotherapy/Screening Service	16	4	Had Surgery	6/8
Napier et al^43^	Canada Public	Prospective service evaluation	47	Shoulder/Knee	Physiotherapy/Screening Service	11	9	Listed for surgery	7/8
O'Farrell et al^44^	Ireland Public	Retrospective service evaluation	50	Shoulder/Knee	Physiotherapy/Screening Service	110	80	Listed for surgery	7/8
Oldmeadow et al^45^	Australia Public	Prospective inter-rater observation	58	Spine/Shoulder/Knee	GPs	38	7	Surgical opinion	7/8
Roland et al^46^	UK Public	Prospective survey	(Not stated)	Mixed	GPs	499	81	Listed for surgery	6/8
Speed and Crisp^47^	UK Public	Retrospective audit	(Not stated)	Mixed	All doctors	362	111	Listed for surgery	5/8
Stilwell et al^48^	NZ Public	Retrospective audit	73	Hip/knee	Physiotherapy/Screening Service	133	87	Listed for surgery	7/8
Walsh et al^49^	Australia Public	Retrospective service evaluation	57	Foot/Ankle	All doctors	72	9	Listed for surgery	7/8
					Podiatry/Screening Service	46	35		
Wood et al^50^	UK Public	Prospective service evaluation	(Not stated)	Spine	Physiotherapy/Screening Service	171	138	Surgical opinion	6/8
							97	Had surgery	

a Neurosurgery referral (remainder are orthopaedic). If only LBP then this is written next to spine. Mixed=more than 3 conditions.

## Results

### General description of studies

**Figure 1.**
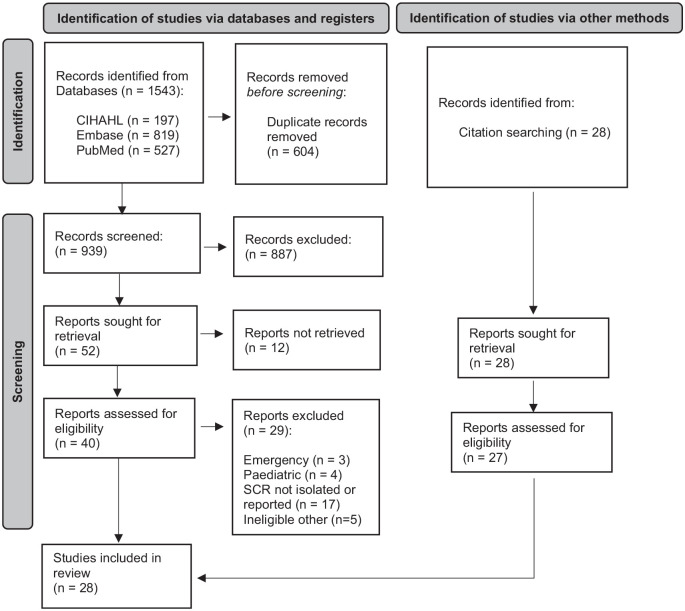
PRISMA diagram.^15^

Twenty-eight studies with a combined total of 5358 patients, met the inclusion criteria (Figure 1). Key information from each study is summarised in Table 1. Three studies included referrals to neurosurgery for spinal pathology,^28,39,40^ while the remainer involved referrals to orthopaedic services. A wide spectrum of musculoskeletal disorders was represented across the included studies; 10 reported surgical conversion rates in cohorts referred with a mixture of musculoskeletal presentations, while the remainder pertained to a specific region or small groups of conditions as detailed in Table 1.

Study quality was generally moderate to high, with scores ranging from 3 to 8 out of a possible total of 8 (Table 1). Full details are reported in the Supplemental File. As all studies reported observational data pertaining to surgical conversion rates, outcome measures were generally well reported. All studies used appropriate statistics for our outcome of interest (SCR). However confounding factors were seldom reported or managed and while discussion of the impact of variable methodologies used to deem patients as surgical (such as opinion vs having surgery) was greatly absent, 3 studies tackled this by simultaneously reporting 2 methods.^36,37,50^

Data pertaining to referrals solely from GPs were extracted from 4 studies, sports physicians in 2 studies, a combined group referred to as *all doctors* in 6 studies and from a screening service in 18 studies (Table 1). All but 2 of these screening services were solely staffed or led by physiotherapists, one involved a combination of physiotherapist and nurse,^32^ one involved a podiatrist.^49^ Two studies presented data from more than one referrer-group, comparing the orthopaedic SCR between a physiotherapy-led screening service and referrals from all doctors,^6^ and another comparing the orthopaedic SCR in referrals from a podiatry screening service compared with referrals from all doctors.^49^ The remaining studies presented data from one referrer-group (Table 1).

### Surgical conversion rates

**Figure 2.**
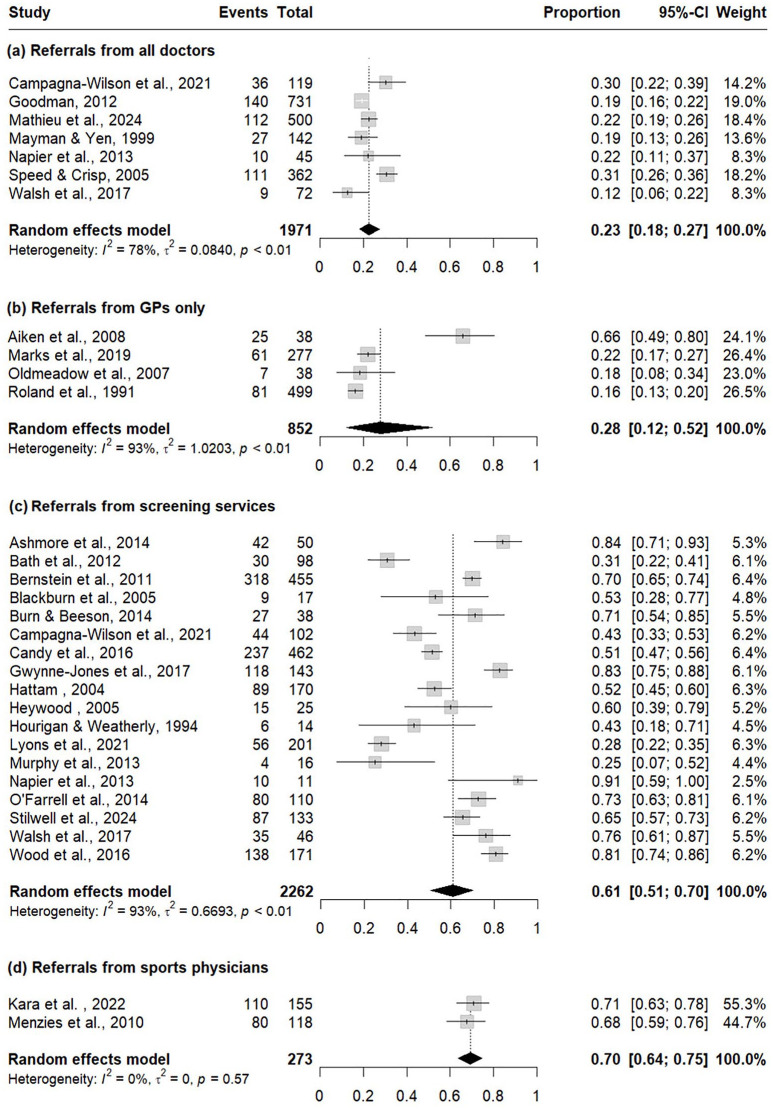
Pooled SCR for referrals from (a) all doctors, (b) GPs only, (c) screening services and (d) sports physicians, using the highest reported SCR from each study out of opinion, listed or had methodologies. Events = surgical cases, Total = total referrals.

Meta-analysis of pooled SCR data, separated into referrer-groups, is presented in Figure 2. Results demonstrate clear differences in orthopaedic SCR according to the referrer. The lowest SCR at 23% was for referrals to orthopaedics from all types of doctors (0.23, 95% CI 0.18-0.27), SCR for referrals from GPs was similarly low at 28% (0.28, 95% CI 0.12-0.52). Higher SCRs were evident from predominantly physiotherapy – led screening services at 61% (0.61, CI 0.50-0.70) and sports physicians at 70% (0.7, CI 0.64-0.75).

### Methodological factors impacting the reporting of SCR

**Figure 3.**
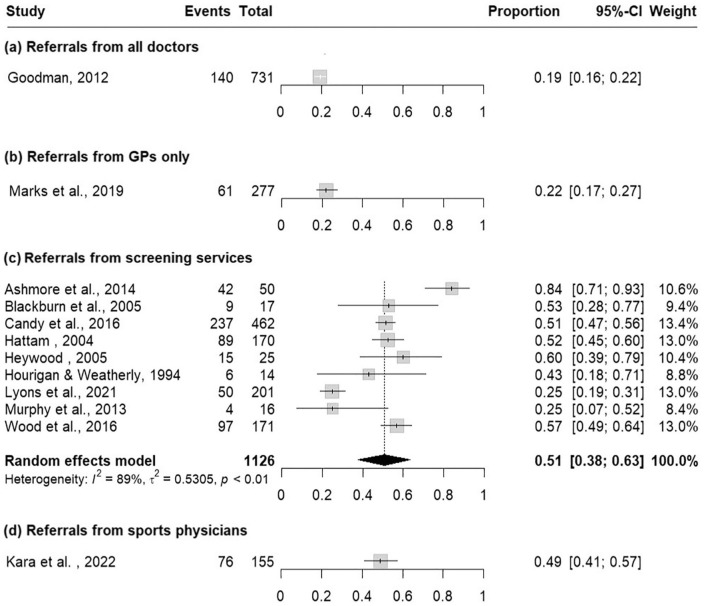
Pooled SCR for referrals from (a) all doctors, (b) GPs only, (c) screening services and (d) sports physicians, using only data from studies reporting SCR in which patients had surgery performed (Had). Events = surgical cases, Total = total referrals.

The methodology used to deem a case surgical varied across included studies. Ten studies reported data in which the surgeon’s opinion was used to deem a case surgical, 9 in which patients were listed/consented for surgery and 12 studies presented data pertaining to cases in which surgery had been performed (Had). While most studies reported only one of these methods, 3 studies used more than one method,^36,37,50^ in each case demonstrating that the SCR is highest when the surgeon’s opinion is used, next highest when surgical listing/consent is used and lowest when actual surgeries performed (Had surgery) is used as the measure to determine surgical cases (Table 1). For this most conservative category of surgery performed (Had surgery), only screening services yielded more than one study; the rate was 51% based on 9 studies (0.51, 95% CI 0.38-0.63), with metanalysis appearing in Figure 3.

## Discussion

This is the first systematic review to comprehensively synthesise musculoskeletal SCR data collated from across the spectrum of referrer types, the first to highlight methodological attributes of research that impact SCR interpretation and the first to present meta-analysis of pooled SCR data. Results demonstrate that the model of care or professional group managing referrals into the surgical unit, has substantial impact upon the SCR in that unit. This highlights that the efficiency of a surgical service is dependent upon potentially multi-professional primary and secondary care musculoskeletal services that support it. Notwithstanding the modest number of included studies and high heterogeneity, pooled observations reveal that when physiotherapy-led screening services (61%) or sports physicians (70%) are responsible for referring patients, orthopaedic SCRs are higher, indicating that the majority of patients referred to surgeons, require surgery. In contrast, when a traditional mix of doctors of all types (23%) or GPs (28%) refer, SCR are lower, indicating that a clear majority referrals do not result in surgery. Furthermore, pooled data for the more conservative measure of SCR, calculated using only data in which surgery was performed, suggest screening services (most often physiotherapy-led) may result in the highest SCR (Figure 3). Higher SCRs are considered desirable,^7^ associated with increased productivity, efficiency and revenue,^8^ better access for patients, potentially better outcomes for patients and reduced societal musculoskeletal economic burden. Consequently, these results suggest that contemporary musculoskeletal service delivery should include models of care that support allied-health/physiotherapy-led referrers and/or more specialised musculoskeletal doctors such as sports physicians.

Previously the SCR from physiotherapy-led screening services had been estimated with a wide range of 25% to 91% in a review of extended scope physiotherapy that retrieved 10 studies reporting SCR.^14^ The present review updates and presents a much richer picture of SCR in physiotherapy-led screening services, including 18 studies reporting SCR for these services, in addition to synthesising the data with meta-analysis and comparing to other referrer types. A variety of factors other than the referrer have potential to impact orthopaedic SCR. Demand side factors may include patient choice and access to community musculoskeletal physiotherapy, while supply side factors may include waiting lists, and patient complexity. Screening services may include multidisciplinary allied health teams, providing a spectrum of physical and lifestyle options that provide patients additional choices in terms of physical activity, diet and psychological care that have been recommended for orthopaedic patients.^51^ However, the impact of such interventions may take time to assimilate into a patient’s complex surgical decision-making framework,^52^ highlighting the need for researchers and service providers to consider how surgery is measured within SCR calculations. It was beyond the scope of this review to examine all these factors. Past reviews into the impact and efficacy of physiotherapy-led triage or screening services have reported the success of these services and also suggested SCRs may be improved by these models of care.^11,12^ The results of this review build on those findings, specifically in relation to SCR. Further research to determine the cost-effectiveness of different referral models is warranted and should include both the cost of referral pathways and the impact of efficiency variations within the surgical unit.

Heterogeneity (*I*^2^) scores were generally high, except in the case of no heterogeneity for sports physicians. As the intention of this review was to present observational data regarding the phenomenon of SCR, rather than trials of treatment effect, consideration of the sources of heterogeneity are more important than its calculated value.^53^ We have identified several key sources of heterogeneity, each of which are important to the understanding of SCR and have not previously been articulated as such in the literature. First is the methodology used to define a case as surgical (opinion, listed, had). Second is the type of referred condition; Table 1 highlights a wide range of conditions across studies. SCRs for different conditions have not previously been investigated and as most included studies did not present data specific to individual conditions, we were unable to undertake secondary analysis of this. Thirdly, the reason for the referral may vary for different studies. This is highlighted by contrasting an included study of 38 referrals specifically for arthroplasty in hip and knee osteoarthritis,^54^ with another included study of 362 mixed referrals for which the referral-reason was unknown.^47^ In the former relatively homogeneous cohort of hip and knee osteoarthritis patients, the SCR might reasonably be expected to be higher than in the latter cohort of mixed conditions, potentially referred for a variety of reasons including diagnostic uncertainty, funding rules etcetera, rather than with the expectation of surgery. Indeed, present understanding of the reasons behind orthopaedic referral represents another gap in the literature, as this has not previously been synthesised and was insufficiently described within included studies in this review, to permit even descriptive analysis. Finally, the professional autonomy of non-medical staff in allied health or physiotherapy-led screening services, creates another potential source of heterogeneity. The number of cases referred to surgeons is likely to be lower, and SCR higher, when allied health staff can autonomously access the necessary imaging and non-surgical care such as medicine prescription and corticosteroid injection. While the UK has made advances in these areas,^55^ internationally access for allied health staff lags behind. In models of care in which allied health staff are required to refer to surgeons to access investigations or non-surgical care (due to traditions or funding rules), SCR will be lower. As the first review to explore the methodological aspects of SCR, we believe these findings are both pertinent to observed pooled results in the present review and highlight methodological considerations for researchers. While the methodological quality of included studies was generally moderate to high, little consideration of the methods used to measure surgical status was encountered and future research into SCR should include details of the referral including the professional responsible, the reason for the referral and clearly articulate the method used to determine surgical cases.

This review has several strengths and limitations. A strength is presentation of the first review to explore the often-used metric of SCR in detail and present a meta-synthesis of data comparing different referrers. A limitation was the variable and often moderate methodological quality of included studies, however this likely had little impact on the primary review aim of reporting SCRs for different referrers. Due to the modest number of included studies and often small sample sizes within studies, numerical SCR calculations must be interpreted with caution. Furthermore, heterogeneity between studies and the discussed reasons behind this heterogeneity, simultaneously highlight the need to apply caution in generalising findings to all settings, yet also represent a strength of this review for being the first to highlight the importance of considering these methodological aspects in future research and service design. A final limitation was the that it was beyond the scope of this review to explore the numerous potential reasons underlying differences in SCR between referrer types. Further research is recommended to identify and quantify these differences.

## Conclusion

Surgical conversion rates in musculoskeletal services vary according to the type of referrer. Lower SCRs result from traditional pathways in which all types of doctors or GPs refer patients, with higher SCRs achieved when referrals are received from physiotherapy-led screening services or sports physicians. Key methodological factors impacting SCR reporting include how cases are deemed surgical, the type of referred condition and the reasons for referral, all of which should be considered in future research. Findings suggest that musculoskeletal / orthopaedic services seeking efficiency through higher SCRs, should consider including sports physician or physiotherapy-led models of care for referral management.
